# Wnt6 signaling regulates heart muscle development during organogenesis^☆^

**DOI:** 10.1016/j.ydbio.2008.08.032

**Published:** 2008-11-15

**Authors:** Danielle L. Lavery, Jennifer Martin, Yvonne D. Turnbull, Stefan Hoppler

**Affiliations:** Institute of Medical Sciences, Cell and Developmental Biology Research Programme, University of Aberdeen, Foresterhill, ABERDEEN, AB25 2ZD, Scotland, UK

**Keywords:** Wnt, Xenopus, Heart, Organogenesis

## Abstract

Mesodermal tissue with heart forming potential (cardiogenic mesoderm) is induced during gastrulation. This cardiogenic mesoderm later differentiates into heart muscle tissue (myocardium) and non-muscular heart tissue. Inhibition of Wnt/β-catenin signaling is known to be required early for induction of cardiogenic mesoderm; however, the identity of the inhibiting Wnt signal itself is still elusive. We have identified Wnt6 in Xenopus as an endogenous Wnt signal, which is expressed in tissues close to and later inside the developing heart. Our loss-of-function experiments show that Wnt6 function is required in the embryo to prevent development of an abnormally large heart muscle. We find, however, that Wnt6 is not required as expected during gastrulation stages, but later during organogenesis stages just before cells of the cardiogenic mesoderm begin to differentiate into heart muscle (myocardium). Our gain-of-function experiments show that Wnt6 and also activated canonical Wnt/β-catenin signaling are capable of restricting heart muscle development at these relatively late stages of development. This repressive role of Wnt signaling is mediated initially via repression of cardiogenic transcription factors, since reinstatement of GATA function can rescue expression of other cardiogenic transcription factors and downstream cardiomyogenic differentiation genes.

## Introduction

Induction of the heart primordia in all vertebrates occurs in paired regions of cardiogenic mesoderm located in the lateral mesoderm (Raffin et al., 2000; Schneider and Mercola, 2001). This cardiogenic mesoderm includes the precursors of both the muscle forming cells (myocardium) and non-muscle forming cells (e.g. pericardium). In Xenopus, these tissues are located in the marginal zone on either side of the dorsal midline adjacent to the Spemann organizing centre in gastrula embryos. The cardiac primordia migrate to the ventral midline where they eventually fuse. Cardiac muscle differentiation begins around stage 26–27 as detected by the induction of cardiomyogenic specific genes, prior to the formation of the linear heart tube at stage 29 and the beginning of a rhythmic heartbeat at stage 34 (Mohun et al., 2000).

Specification and induction of the primary heart field is associated with the expression of two families of transcription factors in particular, the Nkx2 homeobox-containing proteins, such as Nkx2.3 and Nkx2.5 (Sparrow et al., 2000) and the GATA family of zinc finger-containing transcription factors GATA4, GATA5 and GATA6 (Jiang and Evans, 1996; Patient and McGhee, 2002). Nkx2 and GATA families of transcription factors are part of the transcription factor network regulating heart potential and subsequent differentiation. This network regulates the expression of structural cardiomyogenic genes, such as myosin light chain (MLC2) (Latinkic et al., 2004) and TroponinIc (Warkman and Atkinson, 2004), which encode the molecular machinery required for heart muscle function.

The fundamental steps in heart development are remarkably conserved among vertebrates. For initial induction of cardiogenic mesoderm, inhibition of canonical Wnt signaling by Dickkopf-1 (Dkk-1) and Crescent is a critical step in both Xenopus and chick (Marvin et al., 2001; Schneider and Mercola, 1999, 2001). Experimental xWnt8 and xWnt3A overexpression is able to inhibit cardiogenic marker gene expression (Marvin et al., 2001), however, so far no particular endogenous Wnt ligand has been shown to be required to restrict the size of the cardiogenic mesoderm.

Here we show that Wnt6 regulates heart muscle development in Xenopus embryos during organogenesis stages using the β-catenin-dependent canonical Wnt signal transduction pathway. We show that Wnt6 regulates expression of members of the GATA and Nkx families of the transcription factor network and subsequently the expression of downstream structural genes such as MLC2 and TroponinIc.

## Materials and methods

### Whole-mount in situ hybridization

RNA whole-mount in situ hybridization was performed as previously described (Lavery and Hoppler, 2008) with a hybridization temperature of 65 °C. Probes were linearized and transcribed using the High Yield Megascript kit from Ambion (xNkx2.3 with EcoRI and T7 (Evans et al., 1995); xNkx2.5 with HindIII and T7 (Tonissen et al., 1994); xGATA4 with XhoI and T3 (Jiang and Evans, 1996); xGATA-6B with NotI and T7 (Gove et al., 1997); xMLC2 with BamHI and T7 (Evans et al., 1995); xTroponinIc with NotI and T7 (Drysdale et al., 1994)).

### Immunofluorescence staining for Wnt6 and TroponinT

Detection of xWnt6 and xTroponinT protein localization was as previously described (Lavery et al., 2008).

### Morpholino and RNA injections

xWnt6 and mWnt6 capped RNAs were generated using the mMessage mMachine Transcription kit from Ambion as follows: xWnt6 (GenBank accession no. EU332159) was cloned into the EcoRI and XbaI sites of pCS2+, this was then cut with Asp718 and transcribed with SP6; mWnt6 (Itäranta et al., 2002) was cut with XbaI and transcribed with T7. Morpholino Oligonucleotides (MO) were designed and synthesized by Gene Tools (Wnt6MO1: 5′-GATCTGGACAGGGGCAACATGATGG-3′; Wnt6MO2: 5′-TGGGCAGTTAAGTAAAGGGTCCAAC-3′: Wnt6MO3: 5′-TGGTCTTCAGCGCAATCAAGAGAAG-3′; Control MO: 5′-CCTCTTACCTCAGTTACAATTTATA-3′). All MO and MO/RNA mixes were heated for 3 minutes at 65 °C and placed briefly on ice just prior to needle loading and injection.

### In vitro transcription/translation assay

In vitro translation was performed using the TNT Coupled Transcription/Translation kit from Promega with S35-labeled Methionine (Amersham Pharmacia). 250 ng of either xWnt6 plasmid or FrzA plasmid were translated in the presence or absence of 250 ng of the indicated MO. 2 μl of the TNT reaction was run on a pre-cast 4–12% Bis–Tris SDS PAGE gel (Invitrogen) in MES running buffer (Invitrogen). The gel was dried onto a piece of Whatman paper and exposed to Kodak film overnight.

### Western blotting

Protein extracts and Western blotting was performed as previously described (Lavery et al., 2008). The rabbit anti-chicken Wnt6 primary antibody (Zymed, catalog no. 38-3400) was used at a concentration of 2.5 μg/ml and incubated overnight at 4 °C, the rabbit anti-ERK2 primary antibody (Santa Cruz catalog no. sc-154) was diluted 1:1000 and incubated at RT for 2 h. Anti-Rabbit-HRP secondary antibody (Sigma) was used at 1:3000 to detect both Wnt6 and ERK2. The ECL Western Blotting Substrate from Pierce Biosciences was used for Chemiluminecent detection. The membranes were stripped using the Re-Blot Western Blot Recycling Kit (Chemicon).

### qPCR analysis of cardiogenic gene expression

Total RNA was extracted using the Qiagen Rneasy Mini kit. A total of 15 embryos for each experimental condition were harvested but separated into three separate extractions (5 embryos/tube) and recombined at the end of the extraction. Generation of cDNA was done using the QantiTect Reverse Trascription kit from Qiagen. Total RNA yields were quantified and normalized using a spectrophotometer so that 1 μg of each sample was used per 20 μl cDNA synthesis reaction. qPCR was performed using Dynamo Hotstart SYBR green enzyme from Finnzymes on an Opticon II machine with marker gene-specific primers and annealing conditions (xGATA4: forward 5′-GTGCCACCTATGCAAGCCC-3′, reverse 5′-TAGACCCACCCGGCGAGAC-3′, at 62 °C (Jiang and Evans, 1996); xGATA6B: forward 5′-CAGTCTCGCTGTCAGTGG-3′, reverse 5′-TGAAGGCACTCGCTTCTGAG-3′, at 60 °C; xNkx-2.3: forward 5′-GTGACAGCCAGTCCTTACACC-3′, reverse 5′-GACATGAAGGAACTGGAGTCC-3′, at 60 °C; xNkx2.5: forward 5′-GAGCTACAGTTGGGTGTGTGTGGT-3′, reverse 5′-GTGAAGCGACTAGGTATGTGTTCA-3′, at 62 °C (Ariizumi et al., 2003); xTpnIC: forward 5′-CCTTGCAGAACACTGTCAGC-3′, reverse 5′-CAGATTAACTGCCTTGGAACG-3′, at 62 °C (Ariizumi et al., 2003); xMLC2: forward 5′-GAGGCATTCAGCTGTATCGA-3′, reverse 5′-GGACTCCAGAACATGTCATT-3′, at 60 °C (Small et al., 2005); xODC: forward 5′-GTCAATGATGGAGTGTATGGATC-3′, reverse 5′-TCCATTCCGCTCTCCTGAGCAC-3′, at 60 °C (De Robertis' Laboratory Home Page: http://www.hhmi.ucla.edu/derobertis/protocol_page/oligos2004.pdf). Cardiogenic gene expression was normalized to ODC expression levels.

### Heatshock inducible transgenics

Transgenic embryos were created and sorted as previously described (Amaya and Kroll, 1999; Wheeler et al., 2000). Heat treatments were performed as previously described in Wheeler et al. (2000).

### Treatment of Xenopus embryos with BIO and GATA6-GR

For the experiments with the Wnt/β-catenin signaling agonist BIO (6-bromo-iridium-3′-oxime Meijer et al., 2003; Sato et al., 2004) embryos were left to develop in 0.1 × MMR to embryonic stage 20 (Fig. 5; or the stages indicated in Fig. 3 of the Supplementary data) before treatment with BIO (6 μM to 12 μM as indicated in the Figure legends) or MeBIO as a control.

For the rescue experiments with inducible GATA constructs, embryos were injected with 100 pg of GATA6-GR or GATA4-GR (Afouda et al., 2005) into the marginal zone of both dorsal blastomeres at the four-cell stage. Following injection, embryos were incubated in 3% ficoll solution overnight. Then they were placed in fresh 0.1 × MMR until stage 20 when they were placed in 0.1 × MMR containing 20 μM dexamethasone + 6 μM BIO (or in controls either DMSO (solvent used to reconstitute BIO), 20 μM dexamethasone or 6 μM BIO alone) until stage 32 at which point they were fixed in MEMFA for 1–2 h and stored in methanol for future analysis by whole-mount RNA in situ hybridization.

## Results

In this investigation, we tested the hypothesis that Wnt6 functions to regulate heart development and heart muscle differentiation. The Wnt ligand xWnt6 is expressed in embryonic tissues including the ectoderm overlying the developing heart tissue, the endothelial-endocardium, and the endocardium of the outflow tract and atrioventricular region of the heart at later stages, which correspond to heart cushion formation (Lavery et al., 2008).

**Fig. 1:**
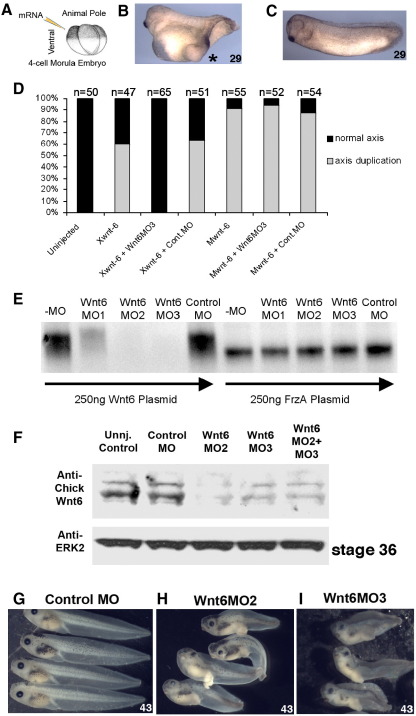
Development of gain and loss of Wnt6 function tools. (A–D) Axis duplication assay after injection of xWnt6 mRNA into one ventral blastomere at the 4-cell stage, as illustrated in panel A. Note formation of an ectopic axis with only 20 pg of xWnt6 mRNA (B, indicated with asterisk; compare to uninjected control in panel C). Note in bar chart (D) of Xenopus axis duplication assays with 75 pg xWnt6 mRNA or 200 pg mWnt6 mRNA that co-injection of xWnt6 MO3 inhibits axis duplication by xWnt6 mRNA but not by mWnt6 mRNA. (E) In vitro transcription and translation assay (TnT); note that xWnt6 protein synthesis (but not control FrzA protein synthesis) is inhibited by Wnt6-specific morpholino anti-sense oligonucleotides (MO1, MO2, MO3), but not by the non-specific Control MO. (F) Western Blot analysis of endogenous xWnt6 expression in stage 36 Xenopus embryos using an antibody raised against chicken Wnt6. Detection of ERK2 is used as a protein loading control. Note that xWnt6 MO2 and MO3 significantly reduce endogenous xWnt6 expression but have no effect on ERK2 protein levels. (G) Morphology of stage 43 embryos injected into the animal pole at the one-cell stage with 30 ng of Control MO (G), Wnt6 MO2 (H) or Wnt6 MO3 (I). Note almost identical abnormal morphologies in Wnt6 MO2 and Wnt6 MO3 morphants with obvious defects in the heart-forming region, the eye, the intermediate mesoderm, the somites, the gut, the pigment cells and the tail fin.

In order to study the function of Wnt6 in Xenopus development, we developed reagents for gain- and loss-of-function experiments. We first tested the molecular activity of full-length xWnt6 cDNA (GenBank accession no. EU332159, Lavery et al., 2008) by injecting xWnt6 mRNA ventrally in early Xenopus embryos (4-cell stage), which lead to axis duplication (Figs. 1A–D; as previously shown for mouse Wnt6 (Itäranta et al., 2002)). This is a functional assay confirming a fully active molecule, but also providing evidence that xWnt6 is capable of inducing canonical Wnt signaling (see below).

**Fig. 2:**
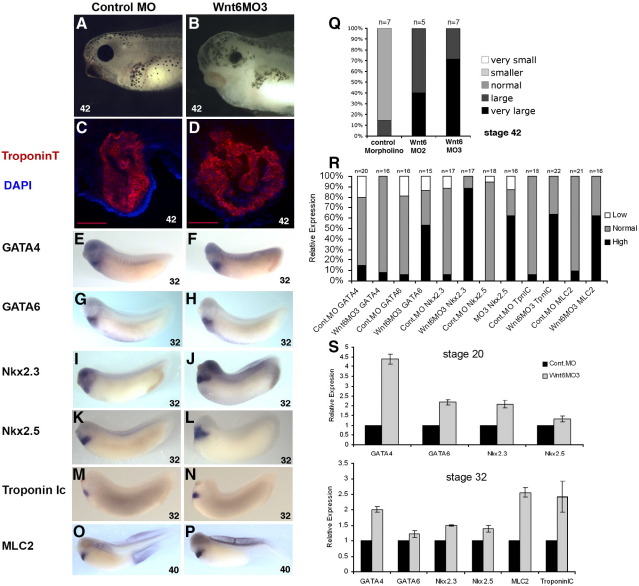
xWnt6 is required for regulating heart muscle development. Morphology of the head and heart forming region at stage 42 (A, B), TroponinT immunohistochemisty analysis of sections through the heart forming region at stage 42 (C, D) and whole-mount RNA in situ hybridization analysis of marker gene expression at stage 32 (E–N) or at stage 40 (O, P) in embryos that were injected into the marginal zone of each blastomere at the two-cell stage with 20 ng of either the Control MO (A, C, E, G, I, K, M, O) or Wnt6MO3 (B, D, F, H, J, L, N, P). Note enlarged tissue mass in Wnt6 morphants in the heart forming region (panel B, compare to panel A) and more TroponinT-expressing myocardial tissue (panel D, compare to panel C, scale bar indicates 100 μm, see also panel Q). Also note slightly stronger and extended expression of GATA4 (panel F, compare to panel E) and GATA6 (panel H, compare to panel G), and much stronger and extended expression of Nkx2.3 (panel J, compare to panel I), Nkx2.5 (panel L, compare to panel K), TnIc (panel N, compare to panel M) and MLC2 (panel P, compare to panel O) (see also panel R). (Q) Percentage bar chart of TroponinT immunohistochemistry analysis of relative size of myocardial tissue in Wnt6 MO- and control MO-injected embryos at stage 42 (see also panels C, D). Note larger TroponinT-expressing tissue in Wnt6 MO-injected embryos. (R) Percentage bar chart of whole-mount RNA in situ hybridization analysis of heart marker expression in xWnt6 MO3- and Control MO-injected embryos at stage 32 (see also panels E–P). Note slightly increased expression of GATA4, and GATA6; and strongly increased expression of Nkx2.3, Nkx2.5, TroponinIc (TnIc) and myosin light chain 2 (MLC2) in xWnt6 MO3-injected embryos compared to controls. (S) Bar chart of qPCR analysis of cardiogenic transcription factor gene expression and heart muscle differentiation markers in xWnt6 MO3- and Control MO-injected embryos at stage 20 and stage 32. Note increased expression of GATA4 in particular at stage 20 and increased expression of heart muscle differentiation markers (MLC2, TnIc) in particular at stage 32 of development.

In order to understand what function xWnt6 may normally play in development; we performed loss of function studies. A knockdown strategy was developed involving anti-sense morpholinos (MO) (see Materials and methods). Three different MOs were tested to examine which would be the most effective and specific. Using an in vitro transcription/translation assay, we demonstrate that all three MOs inhibit xWnt6 translation, with Wnt6MO2 and Wnt6MO3 being the most effective (Fig. 1E). We next tested Wnt6MO2 and MO3 in vivo and found that they both inhibited translation of endogenous xWnt6 as detected by Western blot using a Wnt6 antibody raised against chick Wnt6 (Fig. 1F, for details see Material and methods). Injection of Wnt6MO2 or Wnt6MO3 into Xenopus embryos induced almost identical phenotypes (Figs. 2H, I). Both Wnt6 morpholinos cause morphological defects in the heart forming region (see below), reduced (or in some cases missing) eyes, enlarged fluid filled fins; many suffered from edema; and some had a bent and shortened body axis, as well as defects in gut looping and cloaca formation. This is not seen in embryos injected with Control MO (Fig. 2G). Additionally, we noticed that Wnt6 MO2- or Wnt6 MO3-injected embryos do not swim or respond to physical stimuli (see Movies 1–3 in Supplementary data).

The fact that these two morpholinos cause almost identical phenotypes, despite targeting non-overlapping sequences in the Wnt6 mRNA, argues for a Wnt6 gene-specific knockdown and rules out significant off target effects. We chose to use Wnt6MO3 in most subsequent experiments since it proved more consistent in the strength of its effects on embryos. Axis duplication assays were used to further test the specificity of Wnt6MO3 (Fig. 1D). As expected Wnt6MO3 completely inhibited ectopic axis induction by xWnt6 mRNA, but had no effect on axis induction by mWnt6 mRNA.

### Wnt6 is required for restricting the size of the differentiating heart muscle

The expression of Wnt6 next to and in the developing heart tissue, and the obvious morphant phenotype in the heart-forming region propelled us to investigate a requirement for endogenous Wnt6 function for Xenopus heart development. We used our MO tools to inhibit endogenous xWnt6 expression and analyzed morphological phenotype and expression of marker genes associated with heart development and heart muscle differentiation. We do not detect any obvious early phenotypes, suggesting that xWnt6 is not required during early embryogenesis; either because xWnt6 has no early function or because any early function is redundant with the function of other Wnt genes, or with other mechanisms. Heart formation and function is however clearly disrupted in Wnt6MO2- or Wnt6MO3-injected embryos (Figs. 1H, I; Fig. 2B). The majority of these embryos do not have a heartbeat, or if they do, it is slow or abnormal (see movies 4–8 in Supplementary data). The heart is also more difficult to see than in Control MO-injected embryos, and appears larger and often also in tighter proximity to the endodermal yolk mass (Figs. 1H, I; Fig. 2B). Analysis of TroponinT-expressing heart muscle tissue in Wnt6 morphant embryos shows development of a larger heart with proportionally larger heart muscle tissue (Figs. 2C, D, Q). We detect no evidence for disproportionately thicker myocardial tissue, as previously observed in Wnt11-R morphants (Garriock et al., 2005), which may suggest that Wnt6 is primarily required prior to heart muscle morphogenesis (see below).

When analyzed by whole-mount RNA in situ hybridization at a stage when the heart muscle is differentiating (stage 32), there was a clear up-regulation of many marker genes that are conventionally associated with heart development and heart muscle differentiation (Figs. 2E–N, R). Markers for other tissues were found to be unaffected or even reduced (e.g. the kidney tubule marker NKCC2, data not shown). Loss of xWnt6 function results in a significant increase and expansion of the expression domains of the Nkx2 family members, with Nkx2.3 being expanded more laterally while Nkx2.5 expanded more towards the anterior (Fig. 2L). The cardiomyogenic genes TroponinIc and MLC2 are also induced by loss of Wnt6 function to be expressed stronger and in an extended domain (Figs. 2M–P). Interestingly both GATA4 and GATA6 appear only mildly affected at this late stage (Figs. 2E–H) this is also evident when represented as a percentage bar chart (Fig. 2R).

In addition, quantitative PCR analysis of Wnt6MO3 injected embryos shows a consistent, sequential up-regulation of heartdevelopment associated genes at different stages of development (Fig. 2S). Initially we only looked at stage 20 and stage 32 and from this we can clearly see that at stage 20, expression of both GATA genes is elevated along with Nkx2.3. GATA 4 appears the most elevated (approximately 4.5-fold) with Nkx2.5 only mildly effected. By stage 32 the initial increase of earlier heart markers has begun to lessen, while the later differentiation genes MLC2 and TroponinIc are more than 2-fold elevated. We repeated this qPCR analysis including more developmental stages to get a clearer view of the temporal sequence of effects on these marker genes in response to loss of xWnt6 function (see Supplementary Fig. 1 in supplementary data), which further confirms a sequential deregulation of GATA genes initially during late neurulation, followed by Nkx2 genes and lastly as expected the differentiation markers MLC2 and TroponinIc. GATA genes have been shown to be required for maintenance of Nkx2.5 expression and this early deregulation in GATA4 expression may be responsible for the increase in the expression of the Nkx2 genes (Nemer and Nemer, 2003; Peterkin et al., 2003). Together, the increase in the GATA and Nkx2 genes would then be expected to be responsible for the later induction of the cardiomyogenic genes. Overall, our results show that xWnt6 normally functions to restrict heart muscle differentiation. xWnt6 may be required to prevent heart muscle differentiation in areas of the heart destined to become non-muscle tissue.

### Stage-specific overexpression of xWnt6 inhibits heart muscle differentiation

**Fig. 3:**
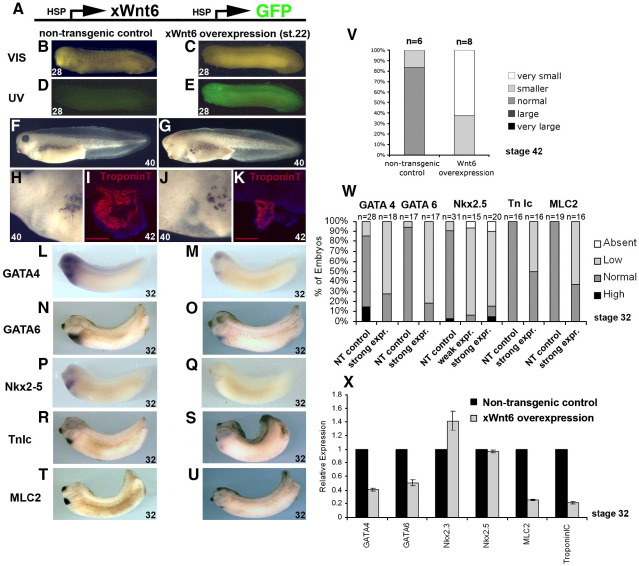
Overexpression of Wnt6 during organogenesis stages inhibits heart muscle development. (A) Schematic representation of transgene for concomitant overexpression of xWnt6 and GFP in transgenic Xenopus embryos. (B-E) Identification of a non-transgenic control embryo (B, D) versus a transgenic embryo (C, E) induced at stage 22 by heat shock treatment to overexpress concomitantly GFP and xWnt6 viewed at stage 28 under UV light (D, E; compare with the same embryos viewed under visible light in panels B and C, respectively). Note only faint background fluorescence (mainly from yolk) in non-transgenic embryo (D), but strong fluorescence due to GFP expression in transgenic embryo (E). External morphology of whole embryos (F, G) and of the heart forming regions of the same embryos (H, J) at stage 40; TroponinT immunohistochemisty analysis of sections through the heart forming region at stage 42 (I, K); and analysis of marker gene expression with whole-mount RNA in situ hybridization at stage 32 (L–U) in non-transgenic control (F, H, I, L, N, P, R, T) and xWnt6-overexpressing transgenic embryos (G, J, K, M, O, Q, S, U). Note abnormal morphology in xWnt6-overexpressing embryos, particularly in the eye (G) and the heart-forming region (G, J). Note much reduced TroponinT-expressing myocardial tissue in xWnt6-overexpressing embryos (panel K, see also panel V). Note significantly reduced and restricted GATA4 (M) and GATA6 (O) expression, dramatic reduction of Nkx2.5 expression (panel Q, but see panel W and panel X) and restricted domains of TroponinIc (TnIc) (S) and Myosin Light Chain 2 (MLC2) (U) expression in xWnt6-overexpressing embryos. (V) Percentage bar chart of TroponinT immunohistochemistry analysis of relative size of myocardial tissue in non-transgenic control and xWnt6-overexpressing transgenic embryos; note much smaller TroponinT-expressing myocardial tissue in xWnt6-overexpressing embryos (see also panels I, K). (W) Percentage bar chart of whole-mount RNA in situ hybridization analysis of heart marker expression in non-transgenic control (NT) and weak or strong xWnt6 overexpression in transgenic embryos. Note reduction of GATA4, GATA6, TroponinIc (TnIc), myosin light chain 2 (MLC2) and generally Nkx2.5 expression in embryos with xWnt6 overexpression. (X) Bar chart of quantitative RT-PCR (qPCR) analysis of heart development marker gene expression in stage 32 embryos. Note reduced expression in xWnt6-overexpressing embryos, apart from Nkx2.3 and Nkx2.5.

The ventral Wnt6 mRNA injection experiments illustrate the effects Wnt6 overexpression has on early embryonic development (see above and Fig. 1). However, endogenous xWnt6 is not expressed at higher levels until organogenesis stages (Lavery et al., 2008), and our loss of function experiments (see above) indicate that xWnt6 is not required until these later stages of development. Wnt6 mRNA injection experiments are unsuitable for studying Wnt function at later stages since the effects Wnt6 overexpression has on early embryonic development would obscure any direct effect Wnt6 might have on later stages of development. In order to study xWnt6 functional activity during organogenesis stages, we used an inducible DNA construct in transgenic Xenopus embryos (see Materials and methods), which allows for stage-specific overexpression of xWnt6 during organogenesis stages (i.e. stage 22), when Wnt6 function was found to be required. The full-length xWnt6 was expressed under control of the Xenopus heat shock protein 70 promoter (Fig. 3A). The transgenic Wnt6-overexpressing embryos developed reduced eyes when compared to the non-transgenic control embryos (Figs. 3F, G), but also an enlarged cavity surrounding a much smaller developing heart (Figs. 3F, G, H, J, see also movies 9–11 in Supplementary data). Analysis of TroponinT-expressing heart muscle tissue shows development of a smaller heart with less myocardial tissue (Figs. 3K, V). The overall shape and morphogenetic looping of the heart is also affected (Figs. 3J, K). When transgenic Wnt6-overexpressing embryos were analyzed by whole-mount in situ hybridization at stage 32, there was a clear reduction in expression of all cardiogenic markers tested, including both genes that are usually associated with cardiac fate specification, such as Nkx2.5, GATA4 and GATA6 (Figs. 3L–Q, W), as well as later heart muscle differentiation genes, such as MLC2 and TroponinIc (Figs. 3R–U, W). Markers for other tissues were found to be unaffected or even up-regulated (e.g. the kidney tubule marker NKCC2, data not shown). The reduction of Nkx2.5, GATA4 and GATA6 expression is not confined to the prospective myocardium tissue but is more general, affecting the wider expression domain of these transcription factor genes (Figs. 3L–Q).

The quantitative effects on gene expression were analyzed by qPCR (Fig. 3X). The majority of the marker genes had reduced expression as a result of xWnt6 overexpression, with the later differentiation genes TroponinIc and MLC2 most affected. These findings confirm that xWnt6 is capable of restricting heart muscle differentiation in Xenopus organogenesis. However, with this analysis both Nkx2.3 and Nkx2.5 expression appear relatively unaffected by xWnt6 overexpression. This may be explained by the few exceptional embryos, which we detect by whole-mount RNA in situ hybridization to have an unusual expansion or up-regulation of Nkx2.5 expression (Fig. 3W).

We also carried out stage-specific xWnt6 overexpression experiments at a stage of development (i.e. stage 14) before we detect strong endogenous xWnt6 expression (Lavery et al., 2008) or defects in our loss-of-function experiments (see above), and analyzed expression of GATA6 and MLC2 at differentiation stages (i.e. stage 32) by whole-mount RNA in situ hybridization (Fig. 2 in Supplementary data). While overexpression of xWnt6 at this earlier stage is clearly capable of restricting heart muscle development, the observed repressive effect is noticeably weaker than when xWnt6 is overexpressed at organogenesis stages (see Fig. 3 and above).

Canonical Wnt signaling has been previously invoked to inhibit heart development during earlier gastrulation stages (Marvin et al., 2001; Schneider and Mercola, 2001), however our experiments show for the first time that a Wnt ligand is capable of restricting heart development at the surprisingly late organogenesis stages. This finding is consistent with the expression of Wnt6 in the embryo (Lavery et al., 2008) and the requirement of Wnt6 function for normal heart development that was revealed in our MO loss-of-function experiments.

### Canonical Wnt signaling mediates regulation of cardiomyogenesis

Since xWnt6 is capable of activating the canonical Wnt signaling pathway as indicated by its ability to induce ectopic axis formation (see above and Fig. 1), we wanted to examine whether canonical Wnt/β-catenin signaling also mediates the effects of xWnt6 during heart organogenesis. We therefore performed stage-specific β-catenin gain-of-function experiments in transgenic Xenopus embryos to assess whether the similar reduction of cardiogenic marker gene expression occurs, as observed with xWnt6 overexpression.

**Fig. 4:**
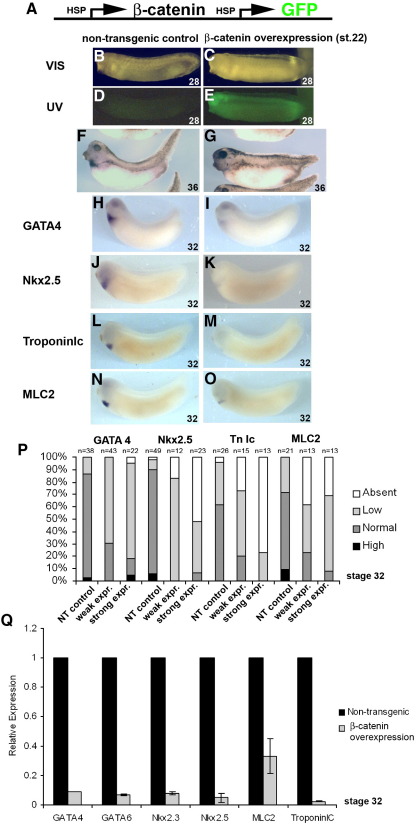
Overexpression of β-catenin during organogenesis stages inhibits heart muscle development. (A) Schematic representation of transgene for concomitant overexpression of a stabilized form of β-catenin and GFP in transgenic Xenopus embryos. (B–E) Identification of non-transgenic control (B,D) versus transgenic embryos (C, E) induced at stage 22 by heat shock treatment to overexpress concomitantly GFP and β-catenin, viewed at stage 28 under UV light (D, E, compare with the same embryos viewed under visible light in B and C, respectively). Note strong fluorescence due to GFP expression in transgenic embryo (E). (F, G) External morphology and TroponinIc expression of non-transgenic control (F) and β-catenin-overexpressing transgenic (G) embryo at stage 36. Note abnormal morphology in β-catenin-overexpressing embryo, particularly the shortened axis and tail, as well as, much reduced TroponinIc expression. (H–Q) Analysis of heart marker gene expression with whole-mount RNA in situ hybridization in non-transgenic control (H, J, L, N) and β-catenin-overexpressing transgenic embryos (I, K, M, O) at stage 32. Note much reduced and restricted GATA4 expression (I), absence of detectable Nkx2.5 (K) and TroponinIc (TnIc) (M) expression and restricted domain of MLC2 expression (O) in β-catenin-overexpressing embryos. (P) Percentage bar chart of whole-mount RNA in situ hybridization analysis of heart marker expression in non-transgenic (NT) control embryos, in transgenic embryos with weak GFP and therefore presumably weak β-catenin expression (weak expr.) and in transgenic embryos with strong GFP and therefore presumably strong β-catenin expression (strong expr.). Note reduction of GATA4, Nkx2.5, TnIc and MLC2 expression in embryos overexpressing β-catenin. (Q) Bar chart of qPCR analysis of heart marker gene expression in stage 32 embryos. Note reduced cardiogenic gene expression in β-catenin-overexpressing transgenic embryos.

Stabilized β-catenin (Yost et al., 1996) was overexpressed in transgenic Xenopus embryos at organogenesis stages (i.e. stage 22) under control of the Xenopus heat shock protein 70 promoter (Fig. 4A). β-catenin-overexpressing embryos develop with a shortened body axis; many also have reduced eyes (data not shown). Overexpression of β-catenin clearly causes reduced expression of all cardiogenic markers tested to an even greater degree than xWnt6 overexpression. The expression of most of the marker genes is clearly reduced and in many cases almost absent in the β-catenin transgenic embryos (Figs. 4H–O). This is also evident when looking at the percent bar charts (Fig. 4P), with the majority of the β-catenin transgenic embryos having reduced or absent expression of cardiogenic genes compared to the control embryos. Markers for other tissues were found to be unaffected or even up-regulated (e.g. the kidney tubule marker NKCC2, data not shown). Quantitative PCR analysis further confirms inhibition of cardiogenic gene expression caused by overexpression of β-catenin and activation of canonical Wnt signaling (Fig. 4Q).

**Fig. 5:**
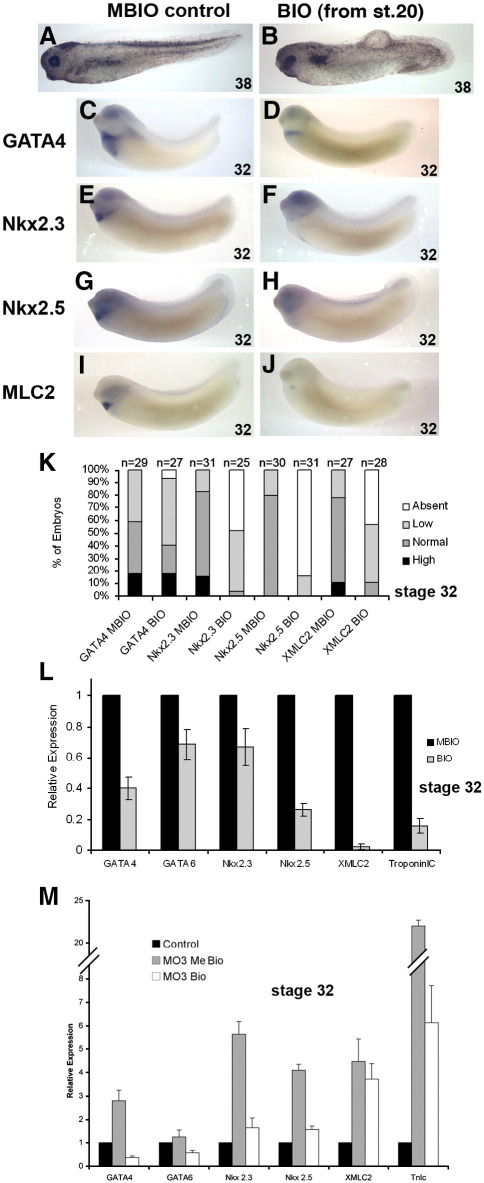
Wnt signaling agonist treatment during organogenesis stages reduces heart marker gene expression, even in Wnt6 morphants. (A, B) Morphology of stage 38 embryos after control treatment with MeBIO (A) or treatment with the Wnt/β-catenin signaling agonist BIO at 12 μM, from embryonic stage 20 to control stage 32. Note multiple morphological defects in BIO-treated embryos in addition to absence of any discernable heart; including enlarged, elongated oval-shaped eyes, which extend toward the anterior; slightly reduced cement glands; reduced pigmentation; a shortened tail; and skin defects. (C–J) Analysis of marker gene expression at stage 32 with whole-mount RNA in situ hybridization in control embryos treated with 10 μM MBIO (C, E, G, I) or experimental embryos treated with 10 μM BIO (D, F, H, J) from stage 20 to 32. (K) Percentage bar chart of whole-mount RNA in situ hybridization analysis of marker gene expression in Wnt agonist-treated (BIO) and control-treated embryos (MBIO). Note that whole-mount RNA in situ hybridization analysis (C–K) shows that all of the marker genes associated with heart development are down-regulated to a various degree by treatment with BIO. (L) Bar chart of quantitative RT-PCR (qPCR) analysis of gene expression of heart development (GATA4, GATA6, Nkx2.3 and Nkx2.5) and heart muscle differentiation markers (TroponinIc and MLC2) in Wnt agonist- and control-treated embryos at stage 32. Note that the heart muscle differentiation markers TroponinIc and MLC2 are most affected along with Nkx2.5 at this stage of development. (M) Bar chart of quantitative RT-PCR (qPCR) analysis of marker gene expression at stage 32 in embryos injected with Wnt6 MO3 and subsequently treated with the Wnt/β-catenin signaling agonist BIO or MeBIO (10 μM from stage 20). Control embryos were injected with control MO and treated with MeBIO. Note that BIO-mediated activation of Wnt/β-catenin signaling during organogenesis stages rescues to a varying extent the deregulated expression of heart development and heart muscle marker genes in Wnt6 morphants.

We alternatively employed the cell-permeable small molecule BIO to activate canonical Wnt/β-catenin signaling at organogenesis stages. BIO has been shown to inhibit GSK3 specifically, stabilize β-catenin and thereby activate canonical Wnt signaling (Meijer et al., 2003; Sato et al., 2004). Treating embryos from stage 20 with BIO results in a fairly severe phenotype (see Fig. 5). The heart in the BIO-treated embryos is difficult to discern (Fig. 5B). Consistent with the xWnt6 or β-catenin overexpression experiments (see above), there is a clear reduction in expression of all heart development-associated marker genes in BIO-treated embryos when analyzed by whole-mount RNA in situ hybridization (Figs. 5C–K) and qPCR (Fig. 5L). To confirm that we were activating canonical Wnt/β-catenin signaling at the optimal time to maximize this inhibitory effect, embryos were treated with BIO at different stages and allowed to develop to differentiation stage 32 for analysis of GATA6 and MLC2 expression by qPCR (Fig. 3 in Supplementary data). Expression of both GATA6 and MLC2 are most affected (down-regulated) by BIO treatment at stages 20–22, consistent with the stage-specific transgenic overexpression experiments (see Figs. 3 and 4).

These experiments show that stage-specific β-catenin overexpression and BIO-mediated activation of canonical Wnt signaling can mediate the effects of overexpressed Wnt6 on heart organogenesis, but only suggest that endogenous Wnt6 signaling acts via Wnt/β-catenin signaling in heart organogenesis. We therefore tested whether the deregulated marker gene expression that we observe as a consequence of knockdown of endogenous Wnt6 in our morpholino experiments (above and Fig. 2) can be remedied to any extent by BIO-mediated activation of Wnt/β-catenin signaling. We find indeed that this artificial activation of intracellular Wnt signaling with BIO treatment from stage 20 reduces marker gene expression in Wnt6 MO3-injected embryos, in the case of the cardiogenic transcription factors close to (i.e. Nkx2.3 and Nkx2.5) or even below (i.e. GATA4 and GATA6) the level of expression in control embryos (Fig. 5M). This result provides much stronger evidence for endogenous Wnt6 to be signaling via the canonical signal transduction pathway.

### Stage-specific activation of GATA can rescue inhibition of cardiogenic genes caused by activation of canonical Wnt signaling

**Fig. 6:**
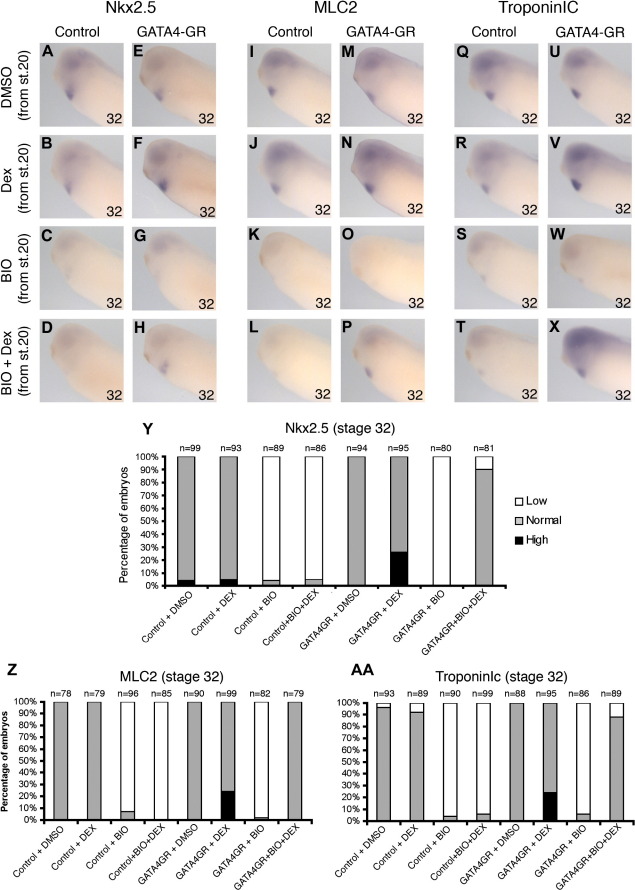
Stage-specific activation of GATA4 during organogenesis stages rescues reduction of cardiomyogenic genes caused by activation of Wnt/β-catenin signaling. (A–X) Analysis of Nkx2.5 (A–H), MLC2 (I–P) and TroponinIc (Q–X) expression with whole-mount RNA in situ hybridization at stage 32 in uninjected (A–D, I–L, Q–T) and GATA4GR-injected (E–H, M–P, U–X) embryos treated from stage 20 with DMSO (A, E, I, M, Q, U); Dexamethasone alone (B, F, J, N, R, V); BIO alone (C, G, K, O, S, W); and BIO and Dexamethasone together (D, H, L, P, T, X). Note in embryos with activated GATA4GR increased Nkx2.5 (F), MLC2 (N) and TroponinIc expression (V); in embryos with BIO-mediated activated Wnt/β-catenin signaling clearly reduced Nkx2.5 (C, D, G), MLC2 (K, L, O) and TroponinIc expression (S, T, W); but note restored Nkx2.5 (H) MLC2 (P) and TroponinIc (X) expression in embryos where BIO-mediated activated Wnt/β-catenin signaling is combined with activated GATA4GR. (Y-AA) Bar charts showing the percentage of embryos with high, normal, or low Nkx2.5 (Y), MLC2 (Z) and TroponinIc (AA) expression in the experiments illustrated in panels A–X (*n* = number of embryos assayed for each treatment).

**Fig. 7:**
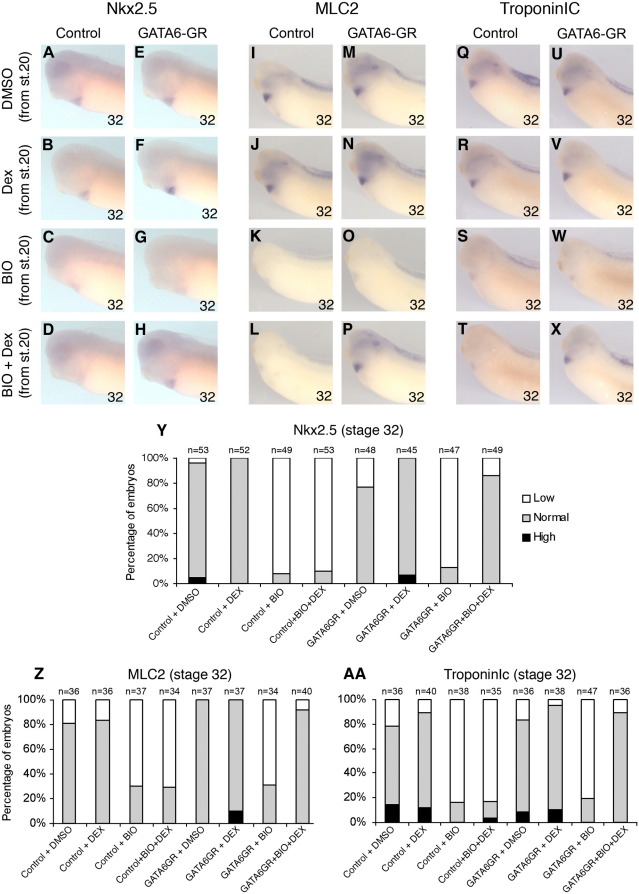
Stage-specific activation of GATA6 during organogenesis stages rescues reduction of cardiomyogenic genes caused by activation of Wnt/β-catenin signaling. (A–X) Analysis of Nkx2.5 (A–H), MLC2 (I–P) and TroponinIc (Q–X) expression with whole-mount RNA in situ hybridization at stage 32 in uninjected (A–D, I–L, Q–T) and GATA6GR-injected (E–H, M–P, U–X) embryos treated from stage 20 with DMSO (A, E, I, M, Q, U); Dexamethasone alone (B, F, J, N, R, V); BIO alone (C, G, K, O, S, W); and BIO and Dexamethasone together (D, H, L, P, T, X). Note in embryos with activated GATA6GR only slightly increased Nkx2.5 (F), MLC2 (N) and TroponinIc expression (V); in embryos with BIO-mediated activated Wnt/β-catenin signaling clearly reduced Nkx2.5 (C, D, G), MLC2 (K, L, O) and TroponinIc expression (S, T, W); but note restored Nkx2.5 (H) MLC2 (P) and TroponinIc (X) expression in embryos where BIO-mediated activated Wnt/β-catenin signaling is combined with activated GATA6GR. (Y-AA) Bar charts showing the percentage of embryos with high, normal, or low Nkx2.5 (Y), MLC2 (Z) and TroponinIc (AA) expression in the experiments illustrated in panels A–X (*n* = number of embryos assayed for each treatment).

In order to investigate mechanisms downstream of Wnt6-mediated regulation of cardiomyogenesis, we tested whether reinstating myogenic transcription factor activity could rescue the expression of cardiomyogenic differentiation markers. We utilized BIO to activate Wnt/β-catenin signaling and dexamethasone-inducible xGATA4 and xGATA6 constructs (xGATA4 and xGATA6-GR, Afouda et al., 2005, which we have recently discovered to rescue cardiogenic development in embryonic explants (Afouda et al., 2008)). As expected, expression of Nkx2.5, MLC2 and TroponinIc were all inhibited by activation of canonical Wnt signaling (Figs. 6G, O, W; Figs. 7G, O, W). However, reinstating either GATA6 or GATA4 activity is capable of rescuing the expression of the other cardiogenic transctiption factor Nkx2.5 and the differentiation markers MLC2 and TroponinIc (Figs. 6H, P, X; Figs. 7H, P, X). There was virtually no rescue in the absence of dexamethasone, confirming the integrity of our inducible GATA constructs. These results suggest that the repressive function of Wnt6 or of canonical Wnt signaling is primarily mediated via reduced expression of cardiogenic transcription factors, such as GATA.

## Discussion

Wnt/β-catenin signaling has previously been inferred to have an inhibitory role during early heart development, because the expression of known Wnt signaling inhibitors such as Dickkopf-1 (Dkk-1) and Cerberus are required for cardiogenic mesoderm induction (Marvin et al., 2001; Schneider and Mercola, 1999, 2001). In this investigation we have identified Wnt6 as an endogenous regulator of heart muscle development, but during the later stages of organogenesis.

Our xWnt6 knockdown experiment shows that xWnt6 is required to restrict heart muscle differentiation. However, deregulation of expression of genes associated with heart development in general and heart muscle differentiation in particular is not limitless. We consider three possible explanations for this finding: first, other Wnt ligands may function partially redundantly with xWnt6 to restrict cardiomyogenesis; second, other molecular mechanisms contribute towards restricting cardiomyogenesis and third, the developmental potential to differentiate into heart muscle is already restricted to some extent in the tissue. We believe there is supporting evidence for any of these three possibilities. xWnt6 is not the only Wnt expressed near the developing heart; Wnt2 is expressed dorsal to the heart-forming region (Landesman and Sokol, 1997) and could therefore contribute towards restricting cardiomyogenesis. However, cardiomyogenesis in this part of the heart field has also been shown to be regulated by other mechanisms, i.e. Notch signaling (Rones et al., 2000) and hence there may be no need to invoke the function of other Wnt ligands in this process. There is also ample evidence that the heart forming potential is restricted to the cardiac mesoderm (or heart field) at stages of development that proceed those during which we demonstrate xWnt6 signaling is required for further restriction of cardiomyogenesis (Garriock and Drysdale, 2003; Rones et al., 2000).

Conventionally, heart development is described as having an early phase when heart potential is induced during gastrulation and a later phase when tissue differentiation proceeds during organogenesis stages. Our overexpression experiments show that Wnt6 is capable of inhibiting heart muscle development relatively late, immediately prior to cardiomyocyte differentiation, which might be taken to suggest that Wnt signaling regulates heart muscle differentiation directly. However, our loss-of-function experiments reveal deregulation of cardiogenic transcription factor gene expression from early organogenesis stages clearly preceding the enhanced expression of structural genes associated with heart muscle differentiation during later organogenesis. Our overexpression experiments always show reduced expression of transcription factor genes, which are usually associated with the regulation of cardiogenic potential. Furthermore, this reduction in expression of cardiogenic transcription factor genes is evident in a wide area of tissue that extends beyond the prospective myocardium. This finding suggests that even at this relatively late stage, Wnt6 primarily regulates cardiogenic potential, which subsequently leads to restricted heart muscle differentiation.

Does xWnt6 also function at earlier stages to regulate the size of the cardiac mesoderm (or heart field)? Our xWnt6 knockdown experiments reveal consequences for heart-specific gene expression and subsequent cardiomyogenesis only from late neurula and early organogenesis stages onwards. Although xWnt6 expression levels are much lower at earlier stages of development (Lavery et al., 2008), our experiments cannot rule out an earlier role for xWnt6 in regulating heart development, which is not revealed in our knockdown experiments because of redundancy with other Wnt ligands or with other molecular mechanisms. Similarly it is likely that Wnt6 has later roles in heart development. We find xWnt6 expression in distinct tissues of the more developed heart, such as the cardiac cushions (Lavery et al., 2008). However, any possible phenotypes in those tissues are difficult to study due to the earlier phenotypes described here resulting from our xWnt6 gene knockdown. Such an additional requirement for xWnt6 function at later stages of heart development may also account for why we often find in our xWnt6 knockdown experiments an enlarged heart that is not properly beating. The Wnt/β-catenin signaling pathway itself has clearly several distinct functions at different stages of vertebrate heart development (Lickert et al., 2002; Marvin et al., 2001; Naito et al., 2006; Schneider and Mercola, 2001; Ueno et al., 2007).

What are the means through which Wnt signaling restricts cardiomyogenesis? We find that deregulated increased GATA gene expression is the earliest response to Wnt6 knockdown and that stage-specific reinstatement of GATA4 or GATA6 function is capable of relieving the repression of cardiomyogenic genes caused by activation of canonical Wnt signaling. This suggests that canonical Wnt signaling is acting at least initially, on regulating expression of cardiogenic transcription factors, even at these relatively late stages just prior to differentiation.

Generally what has been discovered for Xenopus embryonic development proved relevant to the understanding of mammalian embryonic development. Organogenesis stages are also among the most conserved stages of vertebrate development. However to date no expression has been described for Wnt6 in the developing mouse heart; and so far no evidence has been provided for a functional requirement of Wnt6 for mouse heart development.

In conclusion, our investigation shows that Wnt6 functions to regulate heart muscle development. An inhibitory role for canonical Wnt signaling in heart development was previously suspected from the heart development-promoting effect of known inhibitors of Wnt signaling at early stages of development. Wnt6 is the first Wnt ligand shown to be required for restricting heart development. Surprisingly, this requirement is during the later stages of organogenesis immediately prior to differentiation of heart muscle (cardiomyogenesis); and the initial target of regulation even at these advanced stages of heart development are the cardiogenic transcription factors, which were generally thought to regulate cardiogenic cell fate and subsequent heart muscle differentiation.

## Appendix A Supplementary data

Movie 1 Response to physical stimulus at stage 42 in Control Morpholino-injected embryos. Note swimming response to physical stimulus (compare to Movie 2 and Movie 3).

Movie 2 Response to physical stimulus at stage 42 in Wnt6 MO2-injected embryos. Note absence of swimming reaction in response to physical stimulus (compare to control in Movie 1).

Movie 3 Response to physical stimulus at stage 42 in Wnt6MO3-injected embryos. Note absence of swimming reaction in response to physical stimulus (compare to control in Movie 1).

Movie 4 Heart area at stage 42 in Control Morpholino-injected embryo (anterior to the right). Note rhythmic heart beating of normal sized heart (compare to Movies 5–8).

Movie 5 Heart area at stage 42 in Wnt6 MO2-injected embryo (anterior to the right). Note abnormal morphology and abnormal heart beating (compare to control in Movie 4).

Movie 6 Heart area at stage 42 in Wnt6MO3-injected embryo (anterior to the right). Note abnormal morphology and abnormal heart beating (compare to control in Movie 4).

Movie 7 Heart area at stage 42 in Wnt6 MO2-injected embryo (anterior to the left). Note abnormal morphology and almost absence of any detectable heart beating (compare to control in Movie 4).

Movie 8 Heart area at stage 42 in Wnt6MO3-injected embryo (anterior to the right). Note abnormal morphology and almost absence of any detectable heart beating (compare to control in Movie 4).

Movie 9 Heart area at stage 42 in non-transgenic control embryo (anterior to the right). Note rhythmic heart beating of normal sized heart (compare to Movie 10 and Movie 11).

Movie 10 Heart area at stage 42 in transgenic embryo with xWnt6 overexpression at stage 22 (anterior to the left). Note rhythmically beating of smaller than normal heart. (compare to control in Movie 9).

Movie 11 Heart area at stage 42 in transgenic embryo with xWnt6 overexpression at stage 22 (anterior to the right). Note absence of rhythmical heart beating (only faint tremor) in heart area (compare to control in Movie 9).

Suppl. Fig. 1 Sequential increase of cardiogenic marker gene expression in embryos with inhibited Wnt6 expression. (A–H) Bar charts of quantitative RT-PCR (qPCR) analysis of cardiogenic marker gene expression at different stages of embryonic development in Control Morpholino- and Wnt6 MO3-injected embryos. (A–D) Analysis of earlier embryonic stages in one series of experiments, i.e. stage 10 (A), stage 14 (B), stage 18 (C) and stage 22 (D). (E-H) Analysis of later embryonic stages in a separate series of experiments, i.e. stage 20 (E), stage 24 (F), stage 27 (G) and stage 32 (H). Note that cardiogenic gene expression is not considerably increased prior to stages 18-20 in Wnt6 morphant embryos compared to controls, but that the GATA genes, in particular GATA4, appear to be increased earliest, already by stage 18 to 20 (see also Fig. 4). GATA4 expression remains elevated through stage 24 and stage 27 with expression beginning to return to control levels by stage 32. GATA6 expression is also increased consistently at the different stages but it does not appear as affected as GATA4. The Nkx2 genes are not expressed as early as Stage 10 and were only slightly increased at stage 18 to 20 but their expression levels become more elevated by stage 32 when GATA gene expression is returning to control levels. The later cardiomyogenic differentiation marker gene, TroponinIc is actually down-regulated (or delayed) at stage 27 (shortly after its initial expression begins), but is elevated by stage 32 together with the other cardiomyogenic gene MLC2 (see also Fig. 4O). The initial delay in TroponinIc expression may be due to the increased expression of GATA and Nkx2 transcription factors, since heart muscle differentiation does not usually occur until the expression of these early cardiac genes begins to diminish (Gove et al., 1997).

Suppl. Fig. 2 Early overexpression of Wnt6 is capable of inhibiting heart muscle development. (A) Schematic representation of transgene for concomitant overexpression of xWnt6 and GFP in transgenic Xenopus embryos. (B–E) Analysis of GATA6 (B, C) and MLC2 (D,E) marker gene expression with whole-mount RNA in situ hybridisation at stage 32 in non-transgenic control embryos (B, D) and xWnt6-overexpressing transgenic embryos (C, E), induced by heat shock treatment at stage 14. (F) Percentage bar chart of whole-mount RNA in situ analysis of GATA6 and MLC2 gene expression in non-transgenic (NT) control and xWnt6 overexpressing transgenic embryos (as indicated). Note that the domain of GATA6 and MLC2 expression is clearly restricted when Wnt6 is experimentally overexpressed at an early neurogenesis stage (stage 14), but that the reduction of GATA6 and MLC2 expression is less severe than when Wnt6 is overexpressed during an early organogenesis stage (stage 22, Fig. 3).

Suppl. Fig. 3 Activation of Wnt/β-catenin signaling inhibits heart muscle development strongly during early organogenesis stages. Bar charts illustrating GATA6 (A) and MLC2 (B) expression analyzed by quantitative PCR at stage 32 in embryos treated with the Wnt signaling agonist BIO (6µM) at different stages of development (as indicated). Note that activated Wnt/β-catenin signaling at all stages tested, from early neurula to late organogenesis stages, causes subsequent inhibition of expression of the cardiogenic genes GATA6 and MLC2, but that the effect is particularly strong when Wnt/β-catenin signaling is activated in early organogenesis stages; which correspond to the stages when Wnt6 is expressed in tissues close to the developing heart, when we observe early effects on cardiogenic gene expression in Wnt6 loss-of-function experiments and when stage-specifically induced Wnt6 overexpression is also capable of inhibiting subsequent expression of the cardiogenic genes and differentiation of heart muscle tissue.

Supplementary Table 1 Mouse qPCR primer sequences and annealing temperatures.
