# Supplementary figures and images for: Wnt6 signaling regulates heart muscle development during organogenesis
Source: Dev Biol. 2008 Nov 15;323(2):177–88. doi: 10.1016/j.ydbio.2008.08.032 (PMC2593796; doi:10.1016/j.ydbio.2008.08.032)

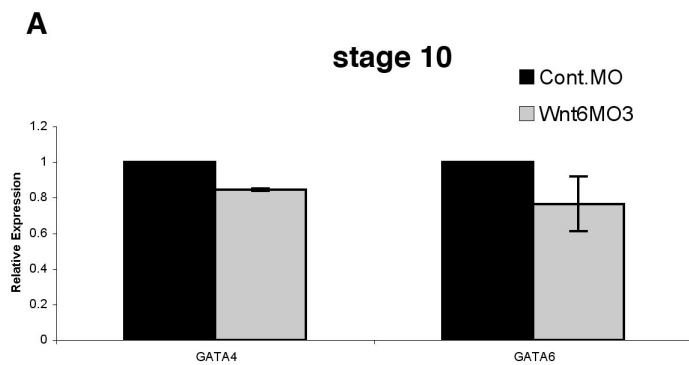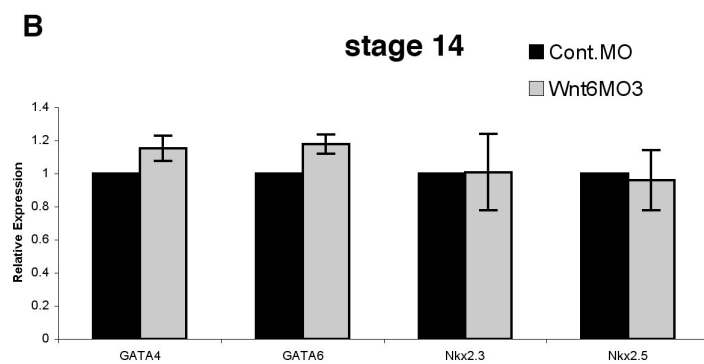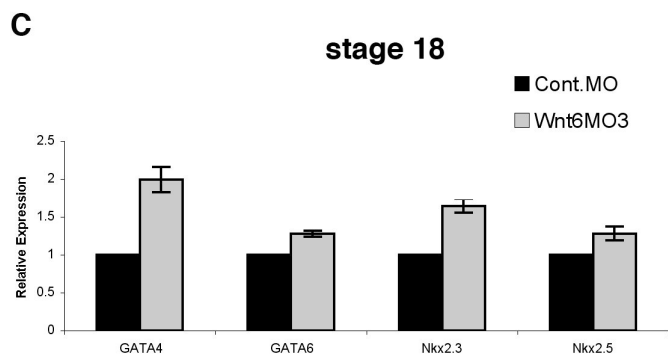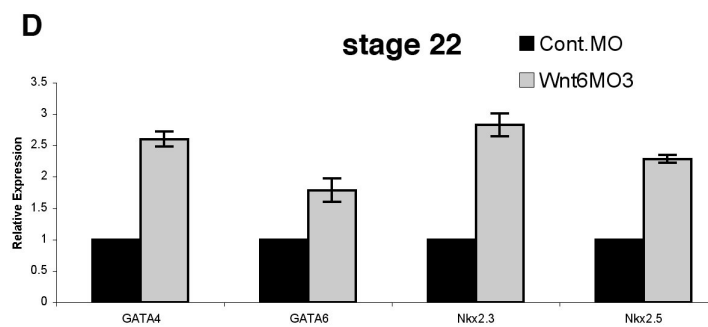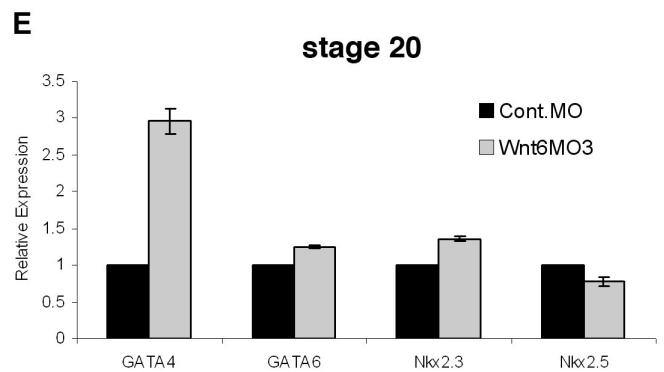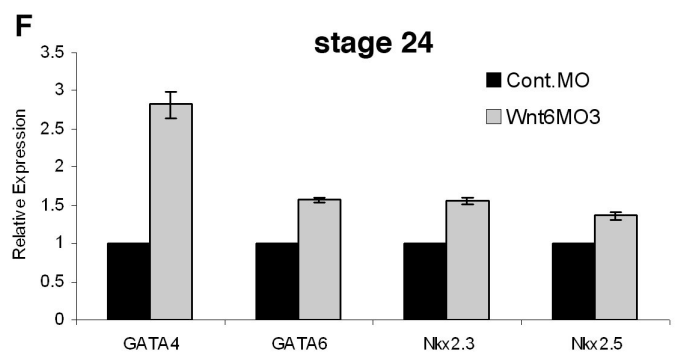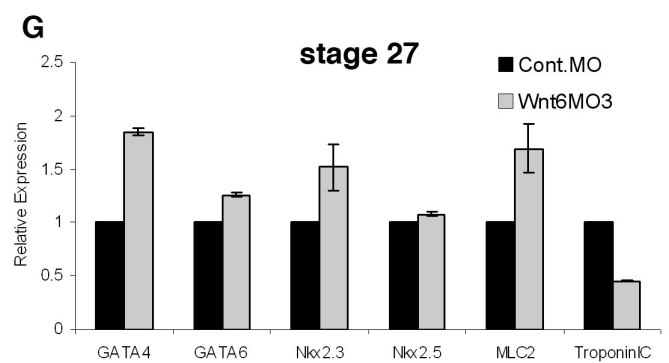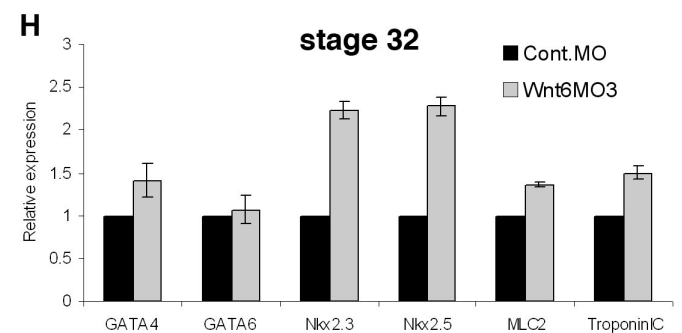

Supplement: Suppl. Fig. 1 — Sequential increase of cardiogenic marker gene expression in embryos with inhibited Wnt6 expression. (A–H) Bar charts of quantitative RT-PCR (qPCR) analysis of cardiogenic marker gene expression at different stages of embryonic development in Control Morpholino- and Wnt6 MO3-injected embryos. (A–D) Analysis of earlier embryonic stages in one series of experiments, i.e. stage 10 (A), stage 14 (B), stage 18 (C) and stage 22 (D). (E-H) Analysis of later embryonic stages in a separate series of experiments, i.e. stage 20 (E), stage 24 (F), stage 27 (G) and stage 32 (H). Note that cardiogenic gene expression is not considerably increased prior to stages 18-20 in Wnt6 morphant embryos compared to controls, but that the GATA genes, in particular GATA4, appear to be increased earliest, already by stage 18 to 20 (see also Fig. 4). GATA4 expression remains elevated through stage 24 and stage 27 with expression beginning to return to control levels by stage 32. GATA6 expression is also increased consistently at the different stages but it does not appear as affected as GATA4. The Nkx2 genes are not expressed as early as Stage 10 and were only slightly increased at stage 18 to 20 but their expression levels become more elevated by stage 32 when GATA gene expression is returning to control levels. The later cardiomyogenic differentiation marker gene, TroponinIc is actually down-regulated (or delayed) at stage 27 (shortly after its initial expression begins), but is elevated by stage 32 together with the other cardiomyogenic gene MLC2 (see also Fig. 4O). The initial delay in TroponinIc expression may be due to the increased expression of GATA and Nkx2 transcription factors, since heart muscle differentiation does not usually occur until the expression of these early cardiac genes begins to diminish (Gove et al., 1997). [file mmc12.pdf]

**A**

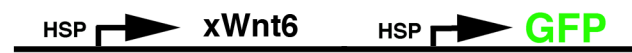

non-transgenic control      xWnt6 overexpression (st.14)

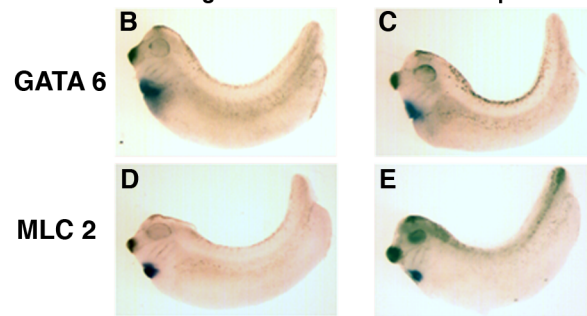

**F**

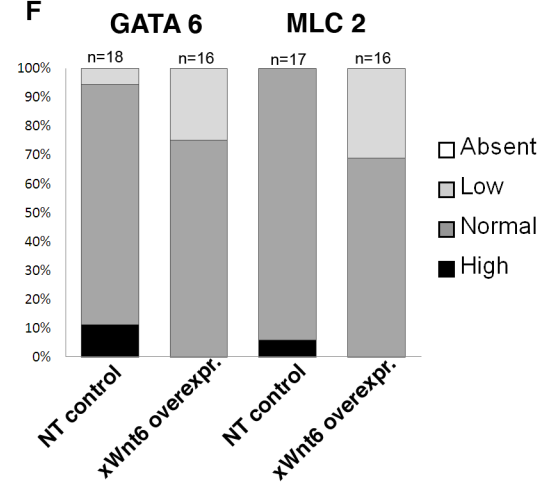

Supplement: Suppl. Fig. 2 — Early overexpression of Wnt6 is capable of inhibiting heart muscle development. (A) Schematic representation of transgene for concomitant overexpression of xWnt6 and GFP in transgenic Xenopus embryos. (B–E) Analysis of GATA6 (B, C) and MLC2 (D,E) marker gene expression with whole-mount RNA in situ hybridisation at stage 32 in non-transgenic control embryos (B, D) and xWnt6-overexpressing transgenic embryos (C, E), induced by heat shock treatment at stage 14. (F) Percentage bar chart of whole-mount RNA in situ analysis of GATA6 and MLC2 gene expression in non-transgenic (NT) control and xWnt6 overexpressing transgenic embryos (as indicated). Note that the domain of GATA6 and MLC2 expression is clearly restricted when Wnt6 is experimentally overexpressed at an early neurogenesis stage (stage 14), but that the reduction of GATA6 and MLC2 expression is less severe than when Wnt6 is overexpressed during an early organogenesis stage (stage 22, Fig. 3). [file mmc13.pdf]

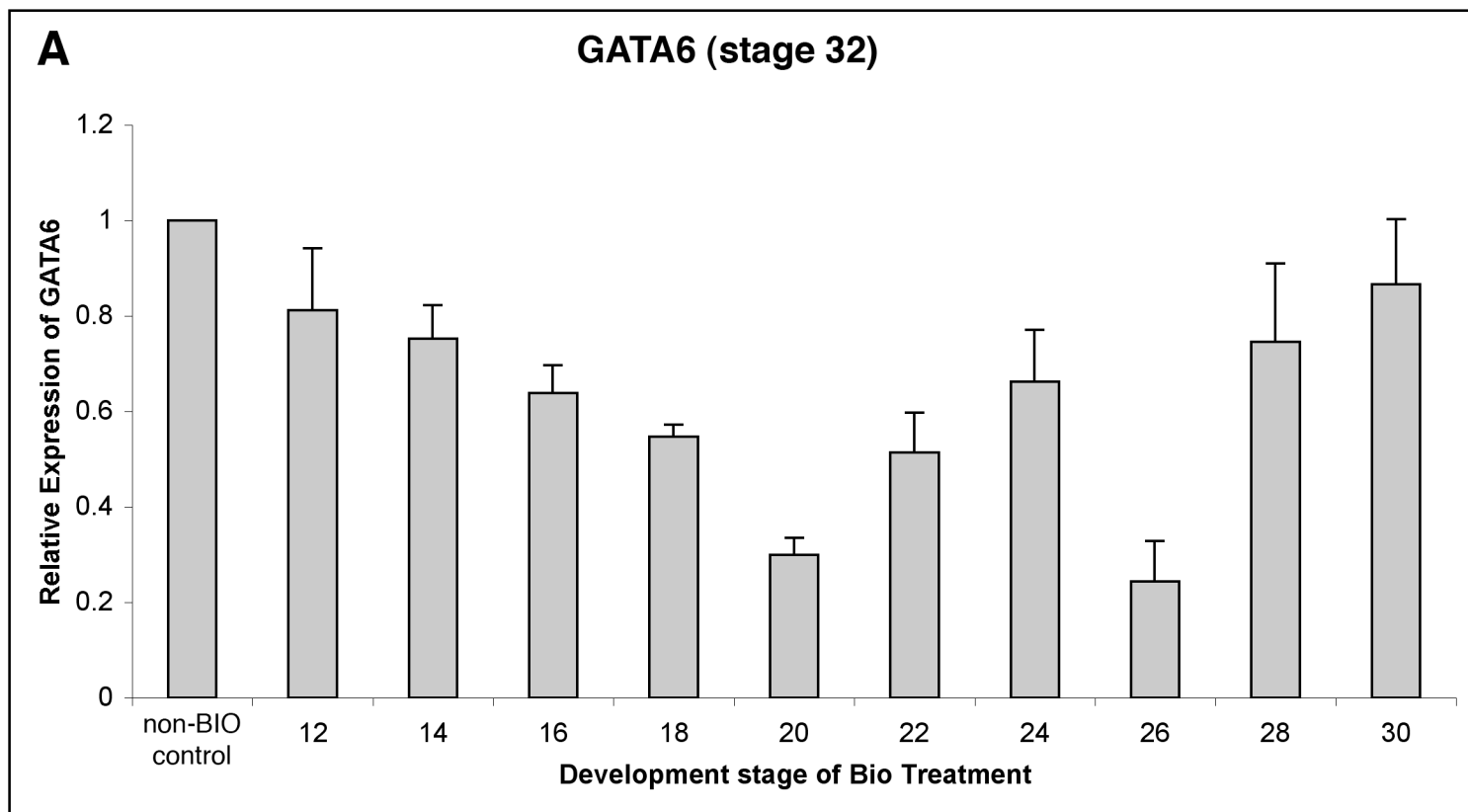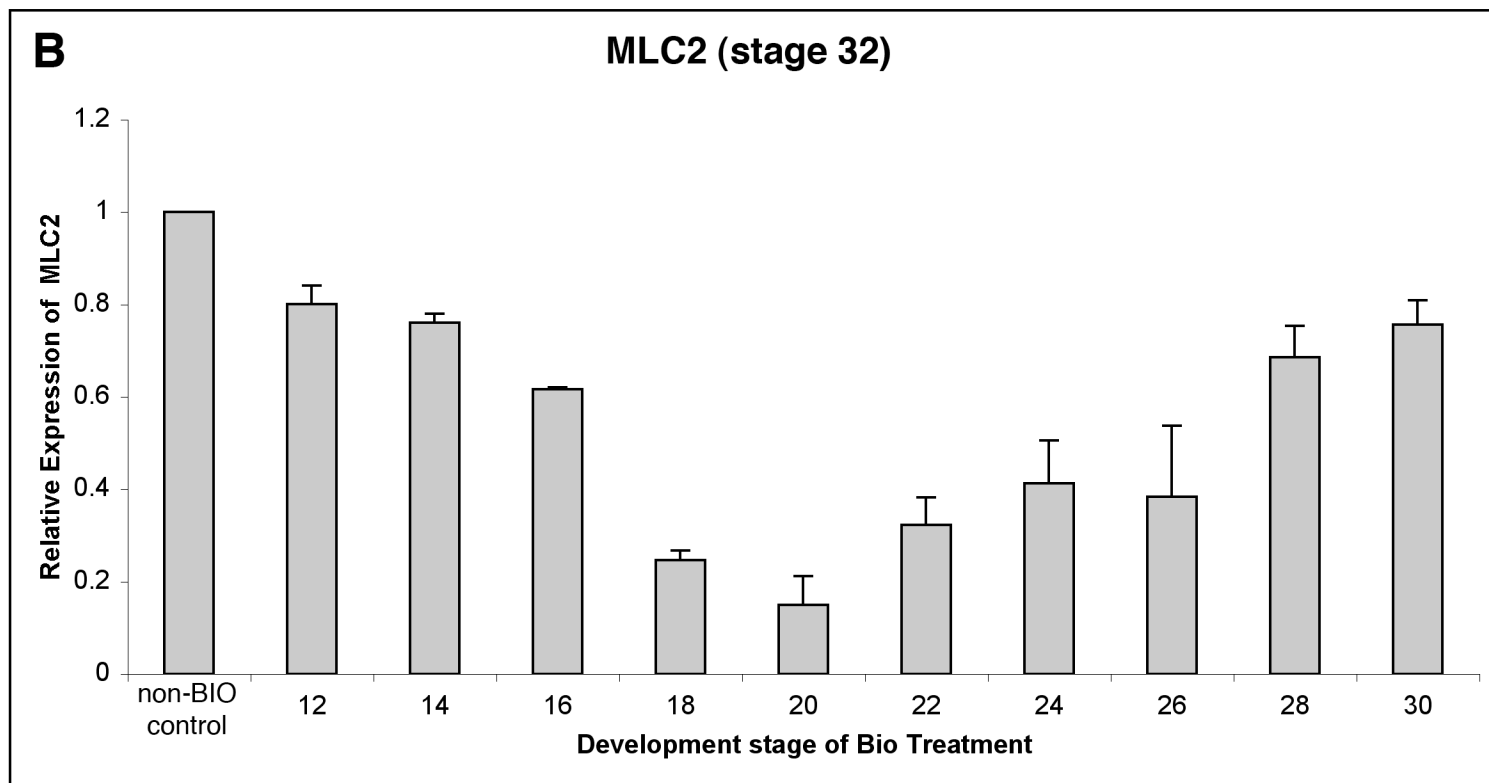

Supplement: Suppl. Fig. 3 — Activation of Wnt/β-catenin signaling inhibits heart muscle development strongly during early organogenesis stages. Bar charts illustrating GATA6 (A) and MLC2 (B) expression analyzed by quantitative PCR at stage 32 in embryos treated with the Wnt signaling agonist BIO (6µM) at different stages of development (as indicated). Note that activated Wnt/β-catenin signaling at all stages tested, from early neurula to late organogenesis stages, causes subsequent inhibition of expression of the cardiogenic genes GATA6 and MLC2, but that the effect is particularly strong when Wnt/β-catenin signaling is activated in early organogenesis stages; which correspond to the stages when Wnt6 is expressed in tissues close to the developing heart, when we observe early effects on cardiogenic gene expression in Wnt6 loss-of-function experiments and when stage-specifically induced Wnt6 overexpression is also capable of inhibiting subsequent expression of the cardiogenic genes and differentiation of heart muscle tissue. [file mmc14.pdf]
