# Supplementary material for: Wnt6 signaling regulates heart muscle development during organogenesis
Source: Dev Biol. 2008 Nov 15;323(2):177–88. doi: 10.1016/j.ydbio.2008.08.032 (PMC2593796; doi:10.1016/j.ydbio.2008.08.032)
Supplement: Supplementary Table 1 — Mouse qPCR primer sequences and annealing temperatures. [file mmc15.doc]

Supplementary Table 1

Mouse qPCR primer sequences and annealing temperatures

| Primer name | Sequence 5'-3' | Annealing temp | Reference |
| --- | --- | --- | --- |
| mTroponin-F2 | GAGTGAGGATCTCTGCAGATG | 60 ^o^C | Lavery, D. L. |
| mTroponin-R2 | CTTCCATGCCACTCAGTGCATC |  |  |
| mNkx2.5-F | GACCAGACTCTGGAGCTTCT | 57 ^o^C | (Liu et al., 2005) |
| mNkx2.5-R | GCGTTGTAGCCATAGGCATTG |  |  |
| mGATA4-F2 | CTCCAGCAATGCCACTAGCAG | 65 ^o^C | Lavery, D. L. |
| mGATA4-R2 | CTGCGATGTCTGAGTGACAGG |  |  |
| mMLC2-F2 | CACAATCATGGACCAGAACAGAG | 65 ^o^C | Lavery, D. L. |
| mMLC2-R2 | CTGCGAACATCTGGTCGATCTC |  |  |
| mWnt6-F2 | GTCACTCAAGCCTGTTCCATG | 62 ^o^C | Lavery, D. L. |
| mWnt6-R2 | GTGCAGTTGCACCAATGCACG |  |  |
| mNanog-F | ATGCCTGCAGTTTTTCATCC | 65 ^o^C | Lavery, D. L. |
| mNanog-R | ACAGTCCGCATCTTCTGCTT |  |  |
| mGAPDH-F1 | GGTCGGTGTGAACGGATTTGG | 65 ^o^C | Lavery, D. L. |
| mGAPDH-R1 | GCCGTGGGTAGAGTCATACTGGAAC |  |  |

qPCR analysis for cardiogenic gene expression was as described for Xenopus (see Materials and Methods) and normalized to GAPDH expression levels.
